# Fabrication and Evaluation of Thermoresponsive GPNMB-Hydrogels as an Innovative Osteogenic Therapeutic Strategy

**DOI:** 10.1007/s11095-025-03978-1

**Published:** 2025-11-18

**Authors:** Tori Czech, Evin Hessel, Jenna Knowles, Kalkedan T. Ameha, Matthew A. Smith, Fayez F. Safadi, Moses O. Oyewumi

**Affiliations:** 1 Department of Pharmaceutical Sciences, College of Pharmacy, Northeast Ohio Medical University, 4209 State Route 44, Rootstown, OH 44272 USA; 2 Department of Biomedical Sciences, College of Medicine, Northeast Ohio Medical University, Rootstown, OH USA; 3 UH-NEOMED Faculty Scholar, Northeast Ohio Medical University, Rootstown, OH USA; 4 Rebecca D. Considine Research Institute, Akron Children’s Hospital, Akron, OH USA

**Keywords:** bone regeneration, fractures, GPNMB, osteoactivin, osteoblasts

## Abstract

**Purpose:**

Earlier studies have reported the ability of GPNMB protein (GPNMB) to promote osteoblast differentiation and function. However, the realization of clinical potential of GPNMB in bone regeneration will require suitable delivery systems to overcome challenges pertaining to poor dosing and poor retention at target sites. Distribution of osteogenic therapeutics away from the desired bone regeneration sites has been linked to serious adverse effects.

**Method:**

We developed thermoresponsive GPNMB-hydrogels using PLA-b-PEG-b-PLA copolymer (10–30% w/v) and demonstrated the ability to undergo solution-to-gel transition at physiologically relevant temperatures. The hydrogel formulations were characterized by vial inversion techniques, dynamic light scattering, rheological assessments and bioretention studies. GPNMB loading (1–10 µg/mL) did not interfere with hydrogel’s thermo-reversibility and viscoelastic behaviors as obtained from rheological strain and frequency sweep tests.

**Results:**

The *in-vitro* release of GPNMB reflected a diffusion-controlled kinetic and is supported by hydrogel degradation pattern involving a rapid loss of the PEG units throughout the 8-week period and a delayed degradation of the PLA units. *In-vivo* long- and short-term safety studies, following GPNMB treatments, showed acceptable serum levels of tissue function and inflammatory markers. There were no detectable signals of ectopic bone formation. Efficacy assessment of GPNMB-hydrogel was based on *in-vitro* osteoblast differentiation and *in-vivo* bone regeneration studies in a murine calvaria defect model.

**Conclusion:**

The biofunctional properties of GPNMB-hydrogels were supported by enhancement of bone regeneration. Additional studies are warranted to fully examine the potential of GPNMB-hydrogel in bone regeneration using a disease model of fracture healing.

**Supplementary Information:**

The online version contains supplementary material available at 10.1007/s11095-025-03978-1.

## Introduction

Bone is a unique tissue that can undergo continuous remodeling while retaining the ability to regenerate itself throughout life [1]. Bone homeostasis is maintained through a harmonious relationship between multiple bone cells. These include osteoblasts (cells that form bone), osteoclasts (cells that resorb bone) and osteocytes (cells that are embedded in bone extracellular matrix). The natural process of bone remodeling is often slow or delayed or ineffective in cases of diseases, trauma and/or injury. Bone disorders affecting the US adult population come in many forms, from a traumatic fracture to gradually worsening osteopenia or osteoporosis [2–4]. In recent years, medical costs for treating bone fractures/diseases have continuously increased [5, 6]. Overall, bone development involves recruitment of osteoprogenitors to the diseased site that later become bone-forming osteoblasts [7]. However, in the case of severe fractures or older populations (with comorbidity), the limited self-healing process cannot adequately help in timely bone repair, leading to functional decline [8, 9]. Application of autografts, allografts and xenografts to treat fractures present challenges such as host rejection, transmission and infection, limiting their extensive use by orthopedic surgeons [10, 11]. Bone tissue engineering using a carrier system incorporated with biomolecules or growth factors can avoid the challenges, promoting bone repair and regeneration [12–14]. There has been great interest in therapeutic alternatives to bone grafting approaches with a prime example in bone morphogenetic proteins (BMPs).

BMPs are a class of biomolecules that induce bone formation by stimulating pluripotent mesenchymal cells to differentiate into osteoblasts [15]. Two recombinant human morphogenetic proteins (rhBMPs) have been extensively studied: rhBMP-2 delivered through a collagen carrier system (INFUSE™) and rhBMP-7 delivered through carboxymethylcellulose/collagen carrier system (OP-1® Putty). Both products have been clinically approved by FDA and investigated in a variety of clinical situations such as spinal fusions, internal fixation of fractures and treatment of bone defects [16–18]. The effectiveness of BMPs in promoting bone regeneration is undeniable but several challenges have limited the appeal and application [19, 20]. These include several grave side effects such as postoperative inflammation, ectopic bone formation and adipogenesis [21]. Considering the low effective dose of BMP-2 in differentiating mesenchymal stem cells to osteoblasts, a supraphysiological dose of 1.5 mg/mL loading onto collagen sponge (INFUSE®) would possibly ensure that there is an effective dose of BMP-2 at site of bone regeneration after an initial burst release from the collagen sponge [22, 23]. Distribution of BMP-2 away from the desired bone regeneration sites has been linked to many adverse effects such as ectopic bone formation (formation of bone in non-target sites) could lead to life- threatening implications [19]. The challenges associated with BMP-2 [22, 23] have sustained ongoing interests in the development of effective and safer alternative therapeutic strategies for healing bone defects.

We consider that glycoprotein nonmetastatic melanoma protein B (GPNMB; osteoactivin) is a viable alternative to BMPs. GPNMB is a type 1 transmembrane glycoprotein (endogenous osteogenic protein) that has been shown to be effective in promoting bone formation [24–28]. The GPNMB cDNA has an open reading frame of 1,716 base pairs and encodes 115 kDa type transmembrane proteins (~ 574 amino acids) that are heavily glycosylated with several functional domains [29, 30]. Earlier studies have found that the recombinant GPNMB protein (rGPNMB; GPNMB protein) which represents the extracellular portion of GPNMB (477 amino acids) can be applied as therapeutic agents in bone regeneration [31–33]. It was reported that GPNMB mRNA and protein are expressed by human osteoblasts while downregulation of GPNMB decreases osteoblast differentiation and function [24]. Further, GPNMB acts as a negative regulator of osteoclast differentiation and survival, which supports the expectation that application of GPNMB protein could potentially be devoid of conflicting bone resorption effects that plagues other osteoanabolic agent such as parathyroid hormone (PTH) and BMP-2 [24, 29, 34, 35]. These findings support the attractiveness of GPNMB as an innovative osteoanabolic therapeutic. Meanwhile, the application of GPNMB therapeutics will require a suitable delivery platform that can enhance its effectiveness, maximize retention at bone fusion sites, reduce or eliminate off-target distribution and effects [36].

Earlier studies applied GPNMB protein adsorbed onto collagen sponge or delivered via gelatin hydrogel [27, 37, 38], here we took another approach to design and develop a delivery system for controlled release of GPNMB-based osteogenic therapeutics, to maximize its effects at bone regeneration sites, which will be vital to realization of its clinical potentials. It is known that the efficacy of bone regenerative growth factors is dependent on the design and application of suitable delivery systems or carriers that can overcome the short half-life and poor retention at the site of action [39–41]. It is particularly desirable that delivery systems/carriers for application in bone regeneration should be stable and biocompatible while effectively achieving sustained release/availability of bone regenerative therapeutics. There is limited information on the safety of rGPNMB as an osteogenic therapeutic agent which was addressed in this work. The studies reported herein aim at fabricating and characterizing thermoresponsive GPNMB-hydrogel formulations while assessing efficacy and safety for potential application in bone regeneration.

Injectable hydrogels are gaining much attention because they can substitute implantation surgery with a minimally invasive injection method and can form any desired shape, to match irregular bone defects [42–44]. Specifically, we considered thermoresponsive hydrogels with the capability of existing as solution at room temperature with phase conversion to gel at physiological temperature [45, 46]. Thus, thermoresponsive hydrogels will make it possible to inject GPNMB therapeutics at room temperature while the subsequent gelation at physiological temperature will facilitate retention at bone fusion sites. In preparing hydrogels, we selected a triblock copolymer poly-(DL-lactide)-b-poly-(ethylene glycol)-b-poly-(DL-lactide), or PLA-b-PEG-b-PLA, as a model of a non-toxic, non-immunogenic, biodegradable copolymer [46]. GPNMB-hydrogels were characterized based on rGPNMB loading, and rheological parameters (loss modulus, storage modulus, strain and frequency sweep, gelation time and temperature). Degradation of the copolymer units used in GPNMB-hydrogel preparation was evaluated by pH build-up, due to release of PLA unit, and matrix-assisted laser desorption ionization (MALDI). *In-vivo* assessments included bioretention and safety studies following perosseous injections of GPNMB-hydrogels in mice. The functional effect of GPNMB-hydrogels was assessed by osteoblast differentiation and the extent of bone regeneration in a murine calvaria defect model. Safety assessment of rGPNMB included serum levels of tissue function and inflammatory markers as well as ectopic bone formation studies. Overall, the work in this report highlighted the fabrication of thermoresponsive GPNMB-hydrogels and evaluation as a new osteogenic therapeutic strategy. The clinical relevance of the work is that GPNMB-hydrogels showed potential of combining impressive safety profiles with efficacy in bone regeneration application.

## Materials and Methods

### Materials

Triblock PLA-PEG-PLA copolymers (1000–1000-1000 Da; 1500–1500-1500 Da; 1700–1500-1700 Da) copolymers and PLA-PEG-PLA (1500–1500-1500 Da) with a covalently attached Flamma Fluor FKR648 endcap were purchased from Akina, Incorporated (West Lafayette, IN, USA). Recombinant GPNMB (rGPNMB; recombinant osteoactivin; rOA) was obtained from R&D Systems (Minneapolis, MN, USA). Phosphate-buffered saline (PBS), and AlamarBlue® reagent, trypsin–EDTA solution at 0.25%, and Gibco® α-Minimum Essential Medium (α-MEM) were obtained from Fisher Scientific (Pittsburgh, PA, USA). Fetal bovine serum (FBS) was obtained from Atlanta Biologicals (Lawrenceville, GA, USA). Trypan blue, Fast Blue RR Salt, Naphthol AS-MX Phosphate solution, formaldehyde solution, and penicillin–streptomycin (Pen/Strep) were obtained from Sigma-Aldrich (St. Louis, MO, USA).

### Fabrication and Characterization of PLA-PEG-PLA Hydrogels and GPNMB-hydrogels

The triblock copolymer, PLA-b-PEG-b-PLA, was tested at varying molecular block weights of 1000–1000-1000, 1500–1500-1500, or 1700 −1500 −1700 Da. These copolymers were utilized in concentrations between 10–40% w/v to be incorporated into water for hydrogel formation using stirring speeds of 700 rpm, at 4ºC. GPNMB-hydrogels were prepared by incorporating various concentrations of rGPNMB (0.1–10 µg/mL) into the PLA-b-PEG-b-PLA hydrogel matrix under gentle stirring for 15 min.

The phase transition behavior of the hydrogels was characterized by a vial inversion method involving maintaining the hydrogel with temperature increases of 1 °C intervals, with temperature equilibration for 10 min [45]. The hydrogel in a scintillating vial was inverted at different temperatures for one minute to observe the fluidity and lack of flow that could signal phase conversion from solution to gel, as signified by no flow within one minute of inversion.

The lowest critical gelation temperature is the temperature at which the solution phase will transition to gel while the upper critical gelation temperature (UCGT) is defined as the temperature at which the polymer begins to precipitate out of the hydrogel system [47]. To determine the LCGT, we applied the vial inversion method approach by initially correcting for heat loss of the hydrogel samples upon removal from heat source for observation. We recorded the time to thermal change from the starting temperature (T0) for each hydrogel formulation. For each T0, time-to-thermal change was performed using a thermometer and stopwatch and recorded as a value of that T0. Then, another data set, time-to-flow, was also recorded for the hydrogels for each T0 upon removal from the heat source, using a stopwatch and observing for any flow of the hydrogel. The two data sets were compared for each T0 and the corrected LCGT was defined as the starting temperature, T0, which had a time-to- thermal change time equal to the time to flow.

The hydrogel gelation time was determined by using vial inversion method for each hydrogel formulation at a time [48]. Briefly, each hydrogel formulation (1 mL per formulation) in scintillation vials was transferred from room temperature into a water bath (maintained at 34 °C as earlier determined as the gelation temperature). By inverting the vial from time to time we captured (using a stop watch) the gelation time at which the hydrogel formed a solid gel. This corresponds to the time at which the hydrogel no longer flows when the vial was inverted [48]. Each assessment was conducted using five replicates.

To assess micelle size and distribution changes of hydrogels during phase transitions and to confirm the proposed gelation mechanism, the size of the hydrogels was measured at varying temperatures using dynamic light scattering (DLS) methods with the Nano-ZS90 Zetasizer (Malvern Instruments, Worcestershire, UK). Solutions of 1 mL of hydrogels were analyzed from 10 °C-40°C, with a 10-min equilibration at each recorded temperature. Transmission electron microscopy (TEM) was applied to visualize the micellar feature of the hydrogels. GPNMB-hydrogels were further characterized by Fourier-transform infrared spectroscopy (FTIR) based on earlier reported methods [49, 50].

Rheological assessment was conducted based on a modified protocol from earlier reports [46]. Briefly, samples were tested using an AR2000 (TA Instruments, New Castle, DE, USA) with a 60 mm (2 degree) cone-plate set up, using a constant oscillatory strain rate of 0.1% applied to the hydrogels, a temperature sweep was conducted from 25 ºC to 42 ºC, by increasing at a rate of 1 ºC/minute. Storage (G’) and loss (G”) moduli were recorded as a function of temperature for each hydrogel as we earlier reported [45]. We also performed rheological strain and frequency sweep tests to assess the viscoelastic behavior of the hydrogel.

### In-vitro Release and Hydrogel Degradation Studies

Dissolution and degradation of hydrogel were carried out by placing hydrogel samples in a physiologically relevant medium while monitoring pH changes over time as well as performing MALDI (matrix-assisted laser desorption ionization and tandem-of-flight) analysis. Briefly, vials containing hydrogels were placed in the New Brunswick Innova 42 incubator shaker (Eppendorf, Enfield, CT, USA) for 5 min at 37 ºC, 0 rpm to allow solidification. Then, isothermal PBS was pipetted on top of the solidified matrix, and the incubator shaker was turned up to 50 rpm (37 °C). The PBS containing the leached polymeric matrix from the hydrogel called leachate, was decanted and replaced at preselected time intervals and both visualization of degradation and pH of decanted PBS (hydrogel leachate) were recorded.

To determine the degradation profile of the copolymers within the hydrogel, samples were prepared for matrix-assisted laser desorption ionization and tandem-of-flight (MALDI-ToF) analysis with freeze drying. Freeze dried PLA-b-PEG-b-PLA polymer and collected hydrogel samples were reconstituted in MeOH (10 mg/mL) and combined with 20 mg/mL of CHCA in MeOH, and 10 mg/mL NaTFA in MeOH, in a ratio of 2:10:1 (v/v), respectively. Then 0.5–1.0 μL of the mixture was spotted onto a 384-well ground steel MALDI target plate and data were obtained using detection range of 0–6000 Da with 1500 shots per sample. Bruker’s FlexAnalysis software was used for data analysis.

To quantify rGpnmb release from the hydrogels, vials containing GPNMB-hydrogels were placed in the New Brunswick Innova 42 incubator shaker (Eppendorf, Enfield, CT, USA) for 5 min at 37 ºC, 0 rpm. Then, isothermal PBS was pipetted on top of the solidified hydrogel matrix and incubator shaker was turned up to 50 rpm (37 °C). Removal and replacement of PBS occurred at different time intervals and analyzed using GPNMB ELISA kit (DuoSet, R&D Systems Inc, Minneapolis, MN). The assay of rGPNMB released was performed in accordance with the directions provided by the manufacturer of the kit. Using Microsoft Excel, we applied data corresponding to 60% of rGPNMB release in model fitting of different kinetic equations such as zero-order, first-order, Higuchi, and Korsmeyer-Peppas [51].

### Cell Culture

MC3T3-E1 (pre-osteoblast) cell line was obtained from ATCC (Manassas, VA, USA). MC3T3-E1 (Pre-OB) cells were maintained in non-osteogenic media: α-Minimum Essential Medium supplemented with 1% (v/v) Pen-Strep (100 µg/mL streptomycin and 100 units/mL penicillin), and 10% (v/v) fetal bovine serum. Cells were maintained in a humidified incubator at 37 °C and 5% CO_2_, and media was changed every 2 days. For osteogenic (+) media, additional supplementation with 0.5% (v/v) β-glycerophosphate and 0.5% (v/v) ascorbic acid was used as described [29, 52]. This media was introduced 24 h after seeding and continued thereafter, with medium being changed every 2 days using our earlier reported method [53].

### In-vitro Biocompatibility and Osteoblast Differentiation Studies

Leachates of hydrogel were collected and diluted in non-osteogenic media prior to incubation with MC3T3-E1 (pre-osteoblast) cell line seeded at 2 × 10^4^ in a 96 well plate. The biocompatibility was analyzed by Alamar® Blue assay using untreated cells as a reference [54]. *In-vitro* efficacy studies with GPNMB-hydrogels were conducted using MC3T3-E1 cells seeded at 4 × 10^5^ per well of a 24 well, HTS Transwell plate. All treatments were introduced into the top compartment of the cell insert and maintained at 37 °C as we earlier reported [45]. Negative control cells were maintained in non-osteogenic media while osteogenic media was applied in osteoblast differentiation studies. The dose of rGpnmb was matched for GPNMB-hydrogel and rGPNMB alone. Media replenishment (without treatment) occurred every two days for 10 and 14 days. Assessment of alkaline phosphatase (ALP) was conducted to determine the extent of cell differentiation induced by the treatments [52, 55]. For ALP assay, we applied Sensolyte® pNPP ALP Assay Kit (Anaspec, Inc., Fremont CA USA) and followed the procedure stipulated by the manufacturer. Results are expressed as ALP activity (ng), normalized to protein assay and compared to positive control cells, cultured with osteogenic media alone, for statistical significance evaluation. For ALP staining, cells were fixed with 4% paraformaldehyde and stained with a solution of Fast Blue RR Salt (Sigma Aldrich, St. Louis, MO, USA) and Naphthol AS-MX Phosphate (Sigma Aldrich, St. Louis, MO, USA) in water.

### In-vivo Retention of Hydrogels

*In-vivo* retention of the hydrogels was conducted using healthy BALB/C mice which were briefly anesthetized with isoflurane and given a 100 µL intramuscular injection of the 10% (w/v) fluorescently tagged hydrogel made with PLA-b-PEG-b-PLA copolymer with Flamma® flour FKR648 endcap (FKR648-hydrogel). Mice were handled in accordance with the guidelines by the Institutional Animal Care and Use Committee (IACUC) at Northeast Ohio Medical University. The *In vivo* Imaging System (IVIS) Lumina XRMS Series III (PerkinElmer, USA) was used to obtain total radiant efficiency of the injection site with an excitation set at 648 nm and emission at 663 nm, over a period of 10 weeks [56, 57]. Time intervals for measurement were preselected at baseline and several time points after injection.

### Safety Assessment of Blank Hydrogel, GPNMB protein and GPNMB-Hydrogel

We followed an approved protocol by the IACUC at Northeast Ohio Medical University. Briefly, C57BL/6 mice (healthy, female, 6–9 weeks) were anesthetized with isoflurane (2%) and given 100 µL of a single, periosseous (intramuscular, bone adjacent) injection in the right lower hindlimb of blank hydrogel, GPNMB-hydrogel or saline. Another set of experiments involved mice receiving a single periosseous injection of rGPNMB solution at a low dose (standard rGPNMB protein dose from literature) [37] of 0.04 µg/g body weight (BW) or high dose 0.2 µg/g BW (5 times higher than the standard dose). After injection, mice were observed briefly and then housed according to the protocols established by IACUC until the predetermined sacrifice time. Serum levels of inflammatory biomarkers were measured by DuoSet® ELISA, according to manufacturer’s protocols. Liver and kidney function assays were also run according to manufacturer specifications. For histological assessment, animals were sacrificed according to approved protocols at prespecified time points. Major organs (liver and kidneys) and injection tissue were harvested, weighed, and then fixed in 10% neutral buffered formalin for 1 week. Subsequently, samples were stored in 30% sucrose at −20ºC before cryosectioning (10 µm sections) with a Leica Cryostat (Leica Biosystems, Buffalo Grove, IL, USA) and staining the sections with hematoxylin and eosin (H&E). Samples were then imaged using a VS120 Scanning Microscope (Olympus, Life Science Solutions, USA) and evaluated for inflammation around the injection site and any major organ damage or abnormalities.

To evaluate potential formation of ectopic bone at the injection site, we conducted dual-energy absorptiometry (DEXA) scans (Lunar PIXImus; GE Healthcare). DEXA scans were carried out on live animals following injections (single, periosseous) of different treatments into the right hindlimbs: blank hydrogel, GPNMB-hydrogel or saline. Scans were completed using X-ray source generating beams at 35 and 80 keV at 0.5 mA and included full body (excluding skull) to monitor weight and total bone development over the assessment period for normalization, as well as right hind limb assessment, with a region of interest defined from toes to spine for each animal. All animals were scanned at baseline for comparison, day 42, and 84 for all treatment groups. After completion of the DEXA analysis on day 84, mice were sacrificed and the hindlimb was harvested from hip to knee and preserved. Micro-CT (µ-CT) analysis was then carried out using a Viva CT 75 µ-CT Scanner (Scanco) at 70kVp, 114μA, slice thickness of 20.5 μm. The preserved hindlimb (hip to knee) was kept with all tissues intact for visualization of any ectopic formation. After µCT analysis, muscle tissue was carefully removed from the femur and was cryosectioned and stained with hematoxylin and eosin (H&E), as previously described, to further identify any ectopic bone formation or inflammatory processes.

### In-vivo Efficacy Assessment of GPNMB-Hydrogel in Bone Regeneration

We applied a calvarial defect model to evaluate the *in-vivo* efficacy of GPNMB-hydrogel in facilitating bone regeneration using healthy C57BL/6 mice (15 weeks; female) as approved by the IACUC at Northeast Ohio Medical University. Briefly, mice were anesthetized, and surgical defects were created with a 2.7 mm trephine (Fine Science Tools, Foster City, CA) on the right side of the calvaria by adapting an earlier reported method [27]. Animals in the sham group did not receive any treatment while the wound area in all treatment groups was layered with absorbable collagen sponge prior to the following treatments: PBS, rGPNMB solution, blank-hydrogel and GPNMB-Hydrogel. The dose of rGPNMB protein in GPNMB-hydrogel and rGPNMB solution was maintained at 0.06 µg/g body weight. Suture closure was performed and vetbond was used to ensure complete wound closure.

Five weeks post operation, animals were sacrificed according to the approved protocols. After sacrifice, calvaria were harvested for analysis. The harvested calvaria were fixed in 4% paraformaldehyde for 72 h at 4 °C before being transferred to 70% EtOH for storage [58, 59]. Using the Skyscan 1273 high-resolution micro-computed tomography machine (micro-CT, Bruker, Billerica, MA) connected to a Hamamatsu L9181-02 camera, high resolution micro-CT scanning was performed on the calvaria at the following settings: 80 kV, 100µA, detection pixel of 10 µm, and 0.5 mm aluminum filter with 2 images captured at every 0.5° through a 360° rotation. The images were reconstructed using Bruker NRecon software and percentage bone volume to tissue volume (BV/TV) were assessed using Bruker CT analysis (CTan) software by creating a 2.7 mm ROI centered on the defect.

### Data Analysis

We applied GraphPad Prism 10 (GraphPad Software, La Jolla, CA) for statistical analysis and data presentation. We analyzed the datasets with student’s t-tests or one-way ANOVA with Dunnett’s or Tukey’s post-hoc tests applied for multiple comparisons. Results are presented as mean ± SD, and differences among groups are considered significant with a p-value < 0.05.

## Results and Discussion

### Fabrication and Characterization of PLA-PEG-PLA Hydrogels and GPNMB-Hydrogels

Hydrogels were successfully fabricated using three different PLA-b-PEG-b-PLA triblock copolymers (10–40% w/v) with varying MW units. These are: Copolymer A: 1000–1000-1000 Da; Copolymer B:1500–1500-1500 Da; and Copolymer C: 1700–1500-1700 Da (Table I). All the copolymers were successfully applied in hydrogel preparation at concentration ranges of 10–30% w/v. We were unable to obtain stable hydrogel formulations at copolymer concentration of 40% w/v possibly due to saturation of the system as revealed by visible amount of unincorporated copolymer and production of highly viscous mixtures (Table I). Among all the copolymers, we found that hydrogel formulations prepared with copolymer A failed to achieve sol-to-gel phase transition at 37 °C (Table I). Meanwhile, copolymers B and C were effective in forming stable hydrogels that displayed sol-to-gel-phase transitions at desirable temperature (37 °C). Formulations made from copolymer B (10–30% w/v) were selected as lead platforms in developing GPNMB-hydrogel based on ease of preparation and thermoresponsive features (Table I). Further, we found evaluated the extent to which the hydrogel thermoresponsive behavior could be challenged with the addition of varying volumes of PBS (10–50% v/v) as part of the loading volume challenge (Supplementary Figure S1).

**Table I:** Preparation of PLA-PEG-PLA Hydrogels at Varying Molecular Weights. Copolymers of Varying Molecular Weight Blocks were Tested at Various Concentrations (10–40% w/v) to Evaluate Successful Hydrogel Formation and Select Formulations to Carry Forward

Copolymer Type	Formulation Rating at Different Co-polymer Concentrations			
	10% w/v	20% w/v	30% w/v	40% w/v
PLA-b-PEG-b-PLA (1000–1000-1000) Da	H	H	H	F
PLA-b-PEG-b-PLA (1500–1500-1500) Da	I	I	I	F
PLA-b-PEG-b-PLA (1700–1500-1700) Da	I	I	I	F

Description of Formulation Rating:

(F) Failed formulations- a stable hydrogel formulation was not formed

(H) Thermally Responsive Hydrogels- the solution-to-gel transition of the hydrogel did not occur at a physiologically relevant temperature

(I) Ideal Hydrogels**-** the solution-to-gel transition occurred at a physiologically relevant temperature

**Fig. 1:**
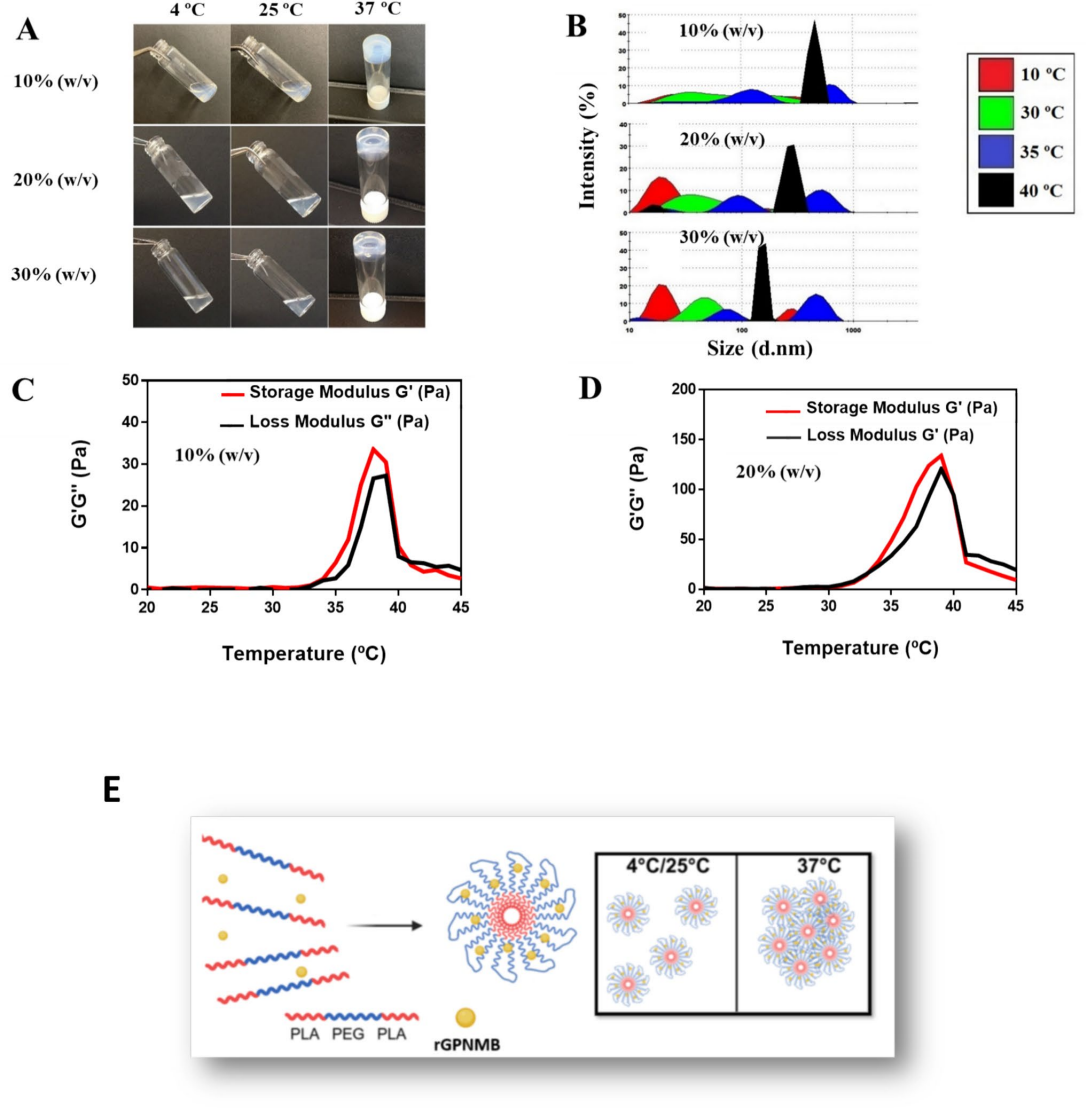
Preparation and characterization of thermoresponsive hydrogel delivery platform. The hydrogels were prepared at various concentrations of the PLA-b-PEG-b-PLA copolymers: 10–30% w/v. **(A)** Representative photographs from vial inversion studies showing the thermogelling behavior of the hydrogel. **(B)** Representative particle size distribution with varying temperature obtained from dynamic light scattering studies. **(C & D)** Representative rheological profiles of the hydrogels at PLA-b-PEG-b-PLA copolymer concentrations of 10% w/v or 20% w/v. At varying temperatures (20–45 ºC), storage modulus (G’) (red) and loss modulus (G”) (black) for each formulation were assessed. **(E)** Schematic illustration of thermoresponsive delivery platform using PLA-b-PEG-b-PLA copolymer and entrapment of rGPNMB protein within the hydrogel matrix.

The sol-to-gel phase transition features for PLA-b-PEG-b-PLA (10–30% w/v) hydrogels were characterized by vial inversion method (Fig. 1A). The light scattering studies showed an increase in the proportions of large sized particles as the temperature increased, irrespective of copolymer concentrations used in hydrogel preparation (Fig. 1B). Our results agree with earlier studies that suggested that the sol-to-gel transition for PLA-b-PEG-b-PLA hydrogels could be attributed to possible formation of aggregates (micelles) as temperature increased [46]. In an aqueous environment, it is expected that the hydrophobic PLA end blocks will fold inward, leaving the protruding hydrophilic PEG block exposed. As the temperature increases, possible changes in the solution-to-gel phase could involve formation of aggregates (micelles) through a rearrangement of the units within the copolymer. We consider that the presence of the hydrogel as a solution at 25 °C will support optimal injectability of the hydrogel into bone defect areas which will eventually undergo phase transition to gel at 37 °C.

We conducted rheological assessments to validate the sol-to-gel phase transition for the hydrogels, particularly capturing the loss and storage moduli as temperature changes [45]. The storage modulus represents the gel-phase behavior while the loss modulus is associated with the solution or liquid phase. Thus, the dominant phase of the hydrogel, at various temperature levels, was observed by capturing the loss (G″) and storage (G′) moduli. At low temperature range (copolymer concentrations of 10–20% w/v) we found low values of both G” and G’ (Fig. 1C-D). As the temperature increased to the transition point, the G′ and G″ of the sample increased sharply to reach a crossover point at which the G′ starts to exceed the G″, indicating an elastic solid-like behavior of the solution beyond the critical temperature point (Fig. 1C-D). The gelation temperature, which is depicted by the cross over of G” and G’, occurred at 34 and 33 ºC, for the 10% (w/v) and 20% (w/v) copolymeric hydrogels, respectively (Fig. 1C-D). As the temperature further increased, the G′ and G″ values continued to increase to 39 ºC for both 10% w/v and 20% w/v copolymeric hydrogels, suggesting the formation of stable hydrogels with certain mechanical stiffness (Fig. 1C-D). Eventually G′ and G″ values decreased after 39 ºC, and again G” became higher than G’ revealing that the hydrogels possibly reversed back to the sol state. A prolonged heat application to the hydrogel most likely facilitated loss of water components and destabilized the hydrogel framework (Fig. 1C-D).

**Fig. 2:**
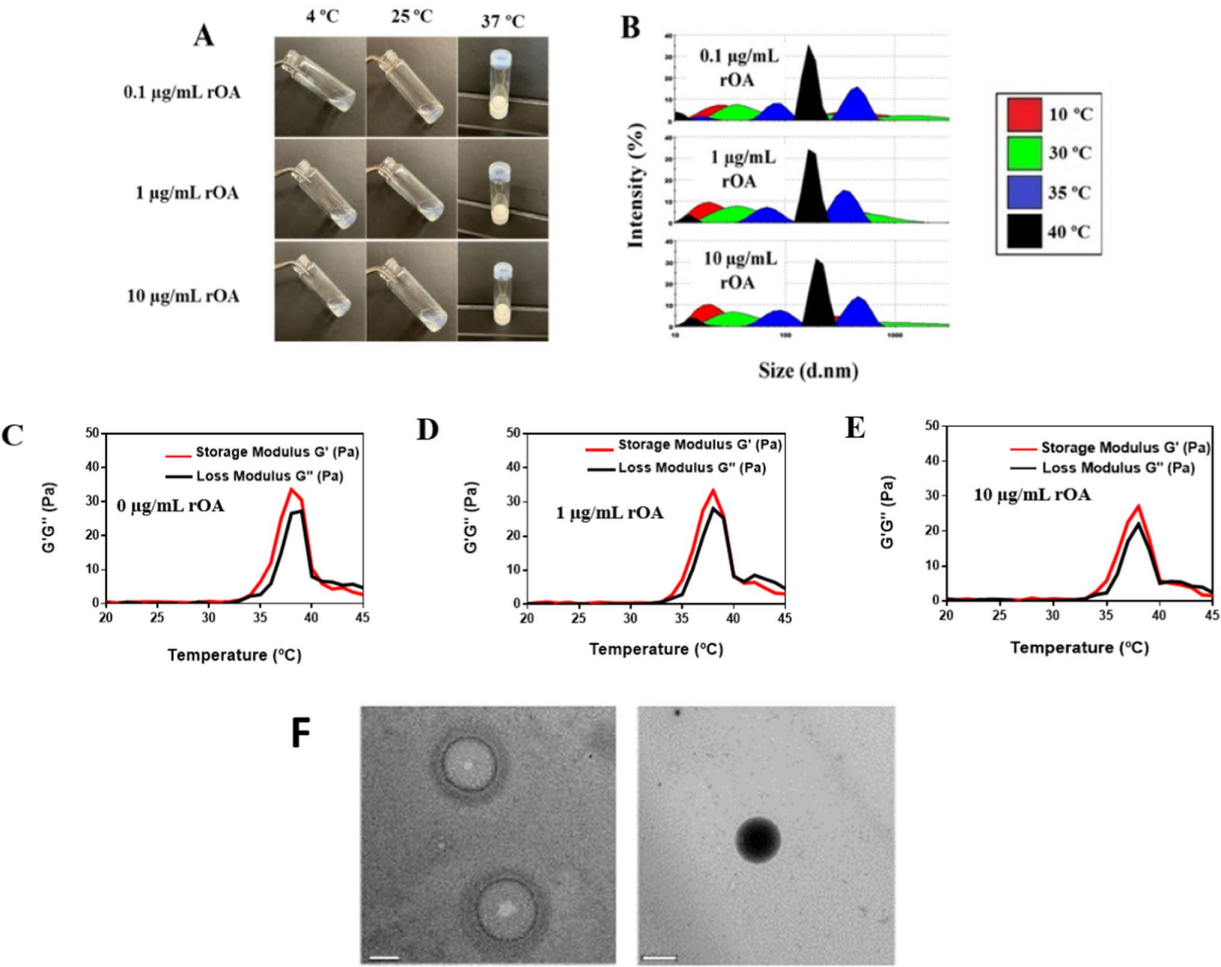
Fabrication and characterization of thermoresponsive GPNMB-hydrogel. **(A)** Representative photographs from vial inversion studies showing the thermogelling behavior of the hydrogels after loading different concentrations of rGPNMB (rOA). **(B)** Representative particle size distribution with varying temperatures of GPNMB-hydrogels as obtained by dynamic light scattering measurements. **(C-E)** Representative rheological profiles showing the storage modulus (G’) (red) and loss modulus (G”) (black) for GPNMB-hydrogels at various rGPNMB (rOA) concentrations. **(F)** Representative TEM images of PLA-b-PEG-b-PLA (blank) hydrogels and GPNMB-hydrogels (10 µg/mL rGPNMB loading). Scale bar is 1 µm.

The general approach to GPNMB-hydrogel fabrication was to load rGPNMB protein within the PLA-b-PEG-b-PLA hydrogel matrix and ensure that the thermoresponsive behavior is preserved through subsequent efficacy studies (Fig. 1E). We successfully formulated GPNMB-hydrogels at rGPNMB concentrations of 0.1–10 µg/mL. The thermoresponsive behavior was measured by the vial inversion and light scattering measurements (Fig. 2A-B). Also, the loss and storage moduli parameters for GPNMB-hydrogel were comparable to blank hydrogels which connotes that rGPNMB loading had negligible effects on the thermoresponsive behaviors of the PLA-b-PEG-b-PLA hydrogels (Fig. 2C-E). We also collected LCGT data (lower critical gelation temperature) for the 20% (w/v) copolymeric hydrogel at rGPNMB loading concentration of 0.1, 1, and 10 µg/mL, showing lower critical gelation temperature (LCGT) of 35 ºC for the blank hydrogel, and 35 °C, 36 °C, and 37ºC, respectively for rOA (rGPNMB) loading concentrations (Supplementary Fig. S2). For hydrogel at 10% (w/v) copolymer, the gelation time, at 34 °C, was found to be 45 ± 5 s by vial inversion method (data not shown). Storage moduli of GPNMB hydrogel (copolymer concentration 10% w/v) at various rGPNMB concentrations indicated that the protein loading did not negatively affect the thermoresponsive behavior of the hydrogel (Supplementary Fig. S3). Overall trend from the vial inversion method and rheological assessments demonstrated the thermoresponsive behavior of GPNMB-hydrogel. The trend agrees with our intention of applying the hydrogel as a liquid for injection into defect sites for subsequent gelation promptly at physiological temperature. Additional rheological characterization (Supplementary Fig. S4) under the strain sweep indicated the viscoelastic properties of the hydrogel where the G’(storage modulus) reflects the elastic behavior while the G” (loss modulus) is associated with the viscous form or energy release due to friction. We observed that GPNMB loading (10 µg/mL using 10% w/v copolymer) did not interfere with the linear viscoelastic region (where the G’ did not change with increasing strain) as depicted by percent critical strain value of 16.15% and 16.20% for blank hydrogels and GPNMB hydrogel, respectively (Supplementary Fig. S4). In the frequency sweep tests, we observed that both the G’ and G” are in close association with each other (G’ > G”) with the values of both moduli increased as frequency increased (0.1–100 Hz) suggesting that the viscoelastic behavior and stability of the hydrogel were maintained (Supplementary Fig. S5). Also, GPNMB protein loading did not affect the trend from frequency and strain sweep tests (Supplementary Fig. S4-S5). Additional studies will be needed to assess the effects of copolymer concentrations on the viscoelastic behavior of GPNMB-hydrogel. Visualization of the matrices of the PLA-b-PEG-b-PLA hydrogels and GPNMB-hydrogel by TEM showed that both formulations have a spherical micellar configuration (Fig. 2F). The staining of TEM micrograph for GPNMB-hydrogel could be indicative of adsorption of rGPNMB protein within the hydrogel micellar structure (Fig. 2F). The FTIR spectra revealed that the wavelength patterns of the blank hydrogel and GPNMB-hydrogel can be nearly perfectly overlaid suggesting that little or no changes to the functional groups as a result of rGPNMB loading (Supplementary Fig. S6). Further, FTIR analysis suggested that rGPNMB loading onto the hydrogel was probably due to surface association on the matrices of the hydrogel with the outer shell (PEG) that is expected to be present on the surface of the copolymeric hydrogel (Supplementary Fig. S6).

### Degradation, Release and Biocompatibility Assessments

**Fig. 3:**
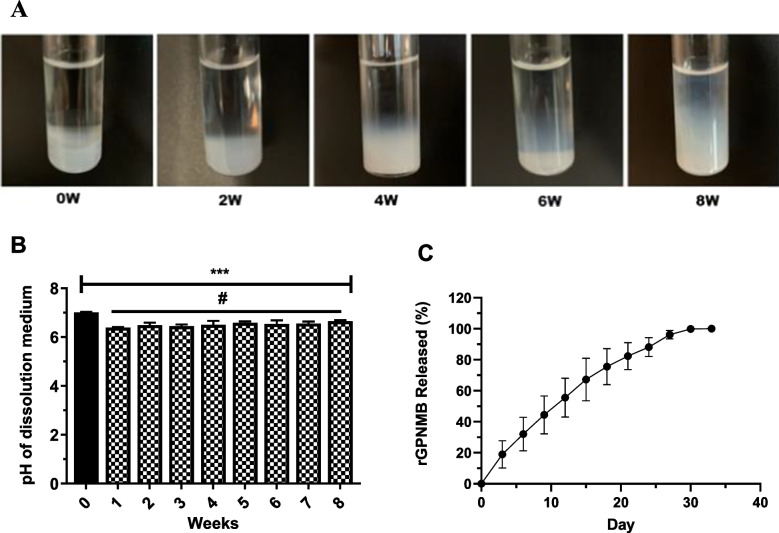
*In-vitro* degradation and release assessment. **(A)** Representative images of PLA-b-PEG-b-PLA hydrogel degradation (37 °C) from week-0 (0W) to week-8 (8W) after preparation. **(B)** The pH of aliquots obtained during PLA-b-PEG-b-PLA hydrogel degradation from week-1 to week-8 after preparation (Each datapoint reflects mean ± SD, n = 4). **(C)** Cumulative release of rGPNMB from hydrogel formulations loaded with (10 µg/ml rGPNMB) as measured by ELISA. Each datapoint reflects mean ± SD, n = 3 independent hydrogel formulations.

Understanding the swelling and degradation behavior of the hydrogels will reflect on the suitability for *in-vivo* application of osteogenic therapeutics. We expect that inferences about the rate, extent, and time for the hydrogel and matrix structures could help in predicting rGPNMB protein release [46, 60, 61]. *In-vitro* degradation studies showed minimal swelling of the hydrogel up to 4 weeks followed by rapid swelling around 6 weeks and an appearance of complete degradation by 8 weeks (Fig. 3A). We proceeded to conduct additional analysis at multiple timepoints during hydrogel degradation using matrix-assisted laser desorption ionization (MALDI). MALDI assesses the distribution of polymers within a sample plotted as the number (intensity) of mass-to-charge (m/z) [62, 63]. Our results show multiple polymer distributions in the high mass region, and a clear decrease in polymer mass over the 8-week period (Supplementary Fig. S7). We noted a biphasic degradation pattern with a rapid loss of PEG units (44 Da repeating units) throughout the entire observation time span while the degradation of the PLA units (72 Da repeating units) was noticeable starting from 6 to 8 weeks (Supplementary Fig. S7).

We measured the pH of the aliquots removed from the degradation of PLA-b-PEG-b-PLA hydrogel due to the possibility that a substantial release of PLA units could cause reduction of pH. Potential risk of lactic acid accumulation in the local tissue area could cause irritation at the injection site [64]. We observed a slight decrease in the pH of the degradation medium (pH 7 to pH 6.5) by the end of week-1, which remained stable around pH 6.5 over the period of 8 weeks (Fig. 3B). The trend suggests that degradation profiles of the hydrogel at copolymer concentration used in the formulation did not cause acid build-up. Overall data from swelling and pH measurements showed that the pH of hydrogel degradation did not become acutely acidic and the platform did not swell excessively, within an initial 4-week period. The trend supports the suitability of PLA-b-PEG-b-PLA hydrogel as a delivery platform for controlled delivery of GPNMB-based osteogenic therapeutics.

Results from *in-vitro* release studies showed that rGPNMB was released in a controlled manner, with no detectable burst release, which persisted for just under 30 days (Fig. 3C). We expect that the rGPNMB release profiles could potentially support bone regeneration or fracture healing by closely matching the time frame for enhanced bone regeneration [65, 66]. Using 60% of rGPNMB release indicated fitting with the zero-order kinetic (R^2^ = 0.994) and Higuchi kinetic equations (R^2^ = 0.988). The R^2^ for Korsmeyer-Peppas and first order kinetic equations were at 0.911 and 0.907, respectively. This is suggestive of a more diffusion-controlled release system [51]. The trend agrees with our observation that the *in-vitro* hydrogel swelling occurred slowly between week-1 to week-4 (Fig. 3A) followed by a period of noticeable copolymer degradation as evident from a significant loss of matrix integrity between week-4 and week-8 (Fig. 3A and Supplementary Fig. S7). Additional studies will be needed to investigate the impact of enzymatic polymer degradation and/or multidirectional release setting for GPNMB-hydrogel [67].

**Fig. 4:**
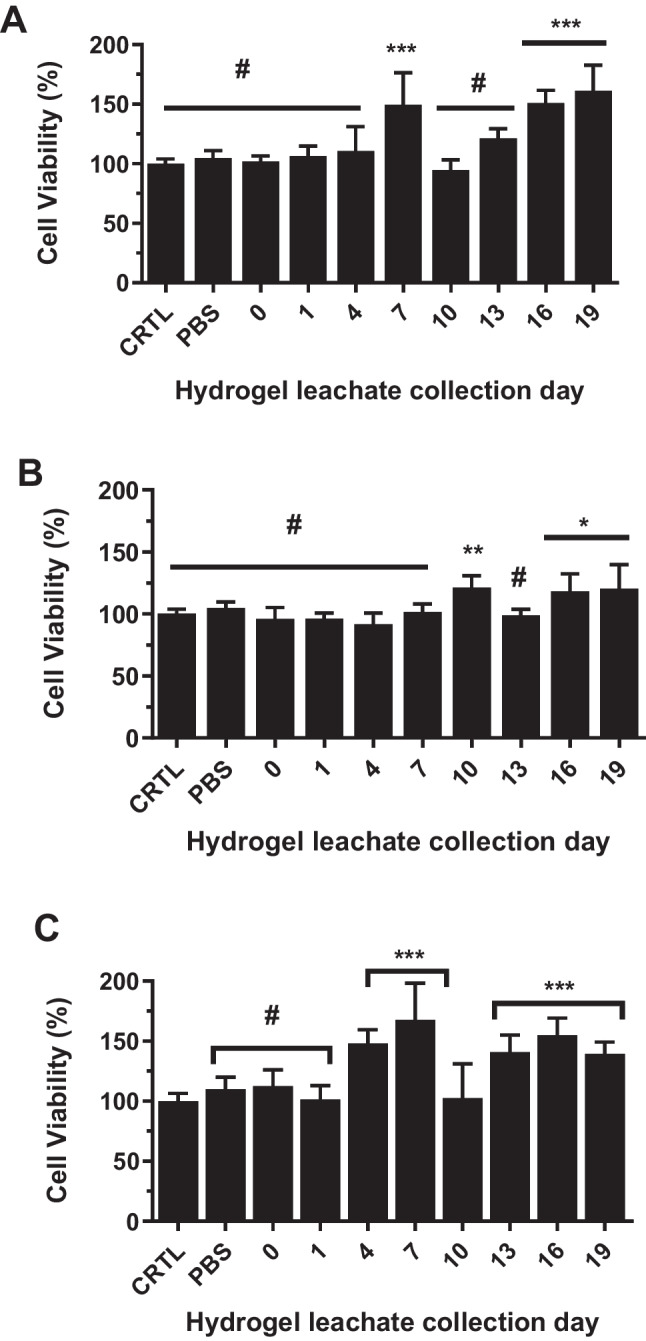
*In-vitro* biocompatibility in pre-osteoblast cells. Effects of leachates obtained from PLA-b-PEG-b-PLA hydrogel degradation (0–19 days) on viability of MC3T3 cells (37 °C; 72 h). The hydrogel leachates were diluted with non-osteogenic cell culture media at ratios (media: leachates) of **(A)** 1:10, **(B)** 1:5 and **(C)** 1:3. #p > 0.05; *p < 0.05; **p < 0.01; ***p < 0.001 *versus* untreated control cells. Each datapoint reflects mean ± SD, n = 6.

Biocompatibility assessments were conducted by incubating (37 °C; 72 h) leachates (aliquots) from PLA-b-PEG-b-PLA hydrogel degradation with pre-osteoblast (MC3T3-E1) cells (Fig. 4A-C). We observed no detectable reduction in cell viability when compared to untreated control cells, indicating that the hydrogel leachates did not have deleterious effects on pre-osteoblast cells (Fig. 4A-C). *In-vivo* hydrogel bioretention study is important to ascertain suitable gelation at physiological temperature as necessary for proper sequestration and confinement of the treatment to injection site. For the bioretention studies, we applied fluorescently tagged copolymeric PLA-b-PEG-b-PLA in making thermoresponsive hydrogel. The retention on the hydrogel after intramuscular injection in mice was monitored by *in-vivo* imaging system (IVIS) (Supplementary Fig. S8) We recorded representative images taken as total radiant efficiency readings from IVIS at select time points over the 10- week observational period. These results show that signals are strong at the earliest time-points, with some continuous, mildly diminishing signal until 6 weeks, at which point signal is rapidly lost (Supplementary Fig. S8). Results obtained show that the retention of the copolymeric hydrogel *in-vivo* is optimal for achieving an enhanced bone regeneration time frame, as fractures take roughly 6 weeks to heal [24]. We recognize that there is the need for follow-up bioretention studies using fluorescently-tagged rGPNMB instead of the copolymer to fully elucidate the ability of the hydrogel delivery system to retain rGPNMB at the fusion site.

### Short- and Long-Term In-vivo Safety Studies

**Fig. 5:**
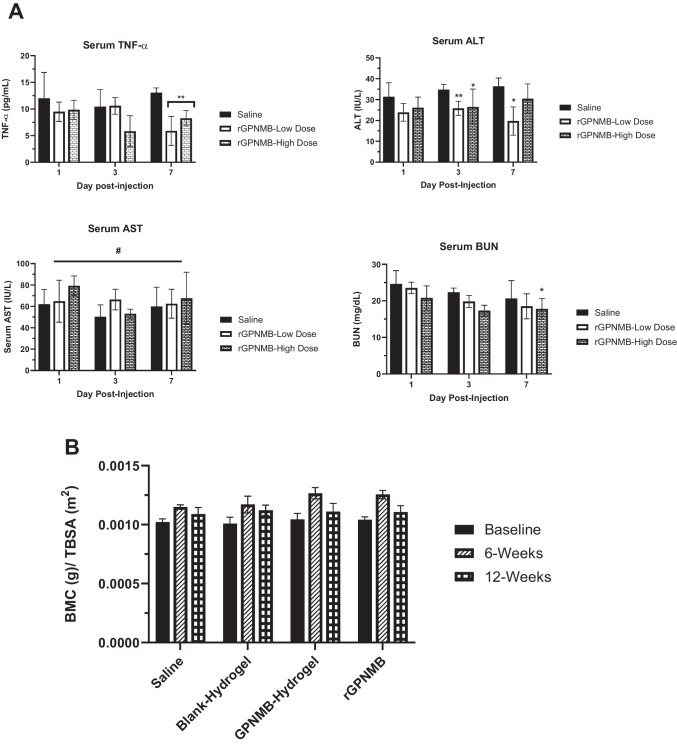
GPNMB protein dose variation and ectopic bone formation. **(A)** Mice (C57BL/6) were monitored until day 7 post injection after receiving periosseous injections of the following treatments- saline, rGPNMB low dose (0.04 µg/g) and rGPNMB high dose (0.2 µg/g). Serum levels of various markers (TNF-α, ALT, AST and BUN) were measured by ELISA at various time points post-injection. Each datapoint reflects mean ± SD, n = 3–4 mice. *p < 0.05; **p < 0.01; #p > 0.05 *versus* saline. **(B)** Mice (C57BL/6) were monitored until day 84 after receiving periosseous injections of the following treatments: saline, blank-hydrogel, GPNMB-hydrogel (0.04 µg/g body weight) and rGPNMB solution (0.04 µg/g body weight). DEXA assessment of BMC of mice at baseline, 6 weeks and 12 weeks post-injection. Each datapoint (mean ± SD, n = 4 mice per group) is reported as BMC normalized by total body surface area (TBSA).

The safety of rGPNMB as a protein therapeutic is not well established. Studies have elucidated the biological pathways with the main focus on understanding the molecular basis of rGPNMB efficacy [24, 29, 34, 35]. However, safety information is critical to any therapeutic development, more importantly for osteogenic agents judging from potential life-threatening challenges with BMPs. Our initial safety assessment was based on rGPNMB dose variation following periosseous injection of rGPNMB solution at two dose levels: rGPNMB-low dose (0.04 µg/g body weight and rGPNMB-high dose (0.2 µg/g body weight). We adopted a 7-day monitoring of key serum parameters and histological assessment of tissue section. We measured serum levels of key markers of inflammation and tissue functions- (a) tumor necrosis factor- alpha (TNF-α) which is a general inflammatory marker [68], (b) alanine transaminase (ALT) and aspartate transaminase (AST) are markers of liver function because these markers are released as a result of liver damage [69], (c) blood urea nitrogen (BUN) is indicative of kidney function in filtering blood by quantifying urea accumulation in the blood [70]. On day-1 post injection, TNF-α level from saline treatment group was comparable to both rGPNMB-low dose and rGPNMB-high dose (p > 0.05; Fig. 5A). We observed that rGPNMB treatment did not cause detectable increase in inflammatory marker, TNF-α. In fact, at the two rGPNMB dose levels, there was a significant reduction in the level of TNF-α on day-7 post injection. However, only rGPNMB-high dose caused a detectable reduction in TNF-α level on day 3-post injection (Fig. 5A). We observed cases of detectable reduction of markers such as ALT on day-3 and day-7 post injection rGPNMB-low dose (p < 0.05, Fig. 5A). The rGPNMB-high dose showed a slight reduction in BUN on day 7 post injection (p < 0.05, Fig. 5A). Overall, both rGPNMB dose levels did not lead to an increase in serum levels of ALT, AST and BUN which possibly indicate no acute detectable kidney or liver injuries (Fig. 5A). Also, we observed no histological changes such as inflammatory cells, cell debris, or other evidence of necrosis in any of the liver and kidney tissue samples harvested post injection of rGPNMB at low and high dose levels (Supplementary Fig. S9).

Another major concern surrounding the use of osteogenic therapeutics is pertaining to the possibility of inducing ectopic bone formation [19, 20]. Ectopic bone formation has been extensively noted with other osteogenic agents such as those of the BMP family [19, 71]. Importantly, ectopic bone formation is a process which occurs over a period of weeks. Thus, we conducted the study in mice that were observed for an extended time period of 84 days after periosseous injection of GPNMB-based osteogenic therapeutics (Fig. 5B). This time period was selected because ectopic bone formation is expected to occur beyond four weeks and may continue to progress in mineralization within this time frame, should it occur [72–74]. As such, we opted to use multiple modalities to monitor the possibility of ectopic bone formation with GPNMB-hydrogels- DEXA and histological approaches. The use of x-ray is notably well suited to assessing mineralized tissue, and is regularly used by researchers and clinicians, alike. The use of x-rays permits the distinction between tissues of differing densities, for example very dense tissues, like mineralized bone tissue, show up opaque (white), while less dense tissues, such as muscle or connective tissue, are nearly transparent (dark) [75, 76]. One approach that makes use of x-rays is dual-energy x-ray absorptiometry (DEXA), this is frequently used in clinical settings to assess the mineralized fraction of bones. This method uses two soft ionization radiation beams to subtract tissue density, giving the measured bone mineral content (BMC) of the tissue [76]. Additionally, DEXA can provide the bone mineral density (BMD) by reporting BMC over the total area of assessment. This approach is extremely useful, as it is a facile method of both bone and ectopic mineralization assessment that is compatible with the use of live animals [74]. Results from the DEXA studies suggest that there was a global increase and then a decrease in parameters such as BMD and BMC over the course of the study. This is consistent with the normal accumulation of bone mass to roughly 17 weeks of age in female C57BL/6 mice, and the subsequent, gradual loss thereafter. Because of this normal occurrence, closely age matching mice was necessary to mitigate variance. Moreover, both BMC and BMD are closely correlated with body weight [76, 77]. Thus, full body scans reporting the weight of the mice were used estimate the total body surface area (TBSA) to further mitigate variation in these parameters as a result of differences in body size among mice [78, 79].

Both BMD and BMC were assessed for each mouse compared to their respective estimated TBSA. However, because BMD takes area into account, it was determined to be a less appropriate measure for this assessment, as our software uses the user-defined region of interest (ROI), which could be dependent on the size of the mouse hindlimb. Instead, BMC was utilized because it is not limited by this constraint and thus provides a more accurate picture of hindlimb mineral content. Therefore, right limb (injection site) BMC was normalized by the TBSA (total body surface area) of the animal and is presented as corrected bone mineral content (BMC). Interestingly, no ectopic bone nodules were visible on the DEXA scans (Fig. 5B). Further investigation with µCT was conducted to scan for any mineralized nodule formation within the local muscle tissue at the site of injection after sacrifice at 84 days for mice treated with saline, blank hydrogel (hydrogel), GPNMB-hydrogel, and rGPNMB at the standard dose (0.04 µg/g body weight) (Supplementary Fig. S10). The representative 3D-µCT images corroborated earlier findings from DEXA that there are no mineralized nodules that would suggest ectopic bone formation present in any of the samples (Supplementary Fig. S10). Additional studies will be needed at varying doses of GPNMB-hydrogel and rGPNMB protein.

**Fig. 6:**
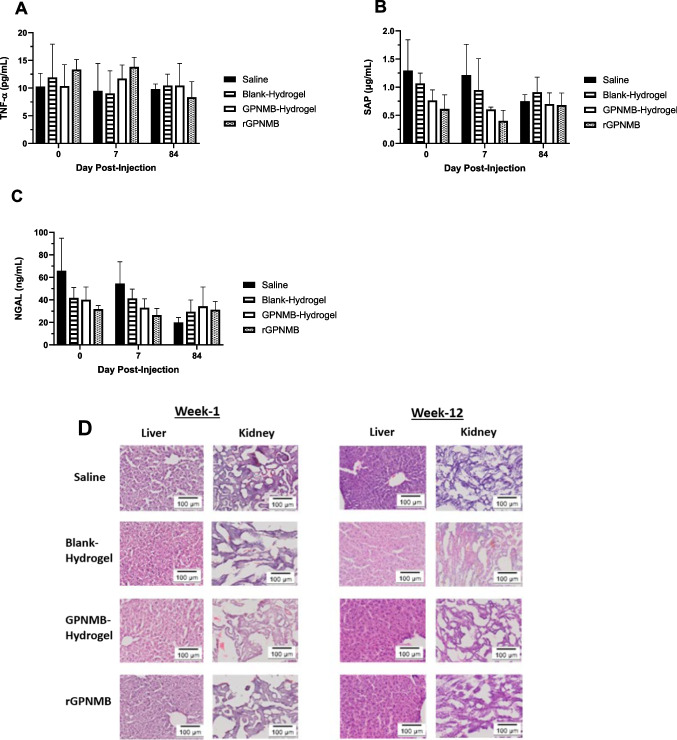
A long-term safety assessment of GPNMB-hydrogel. Blood samples were withdrawn from mice (C57BL/6) at various time intervals till day 84 after receiving periosseous injections of saline, blank-hydrogel, GPNMB-hydrogel (0.04 µg/g body weight) and rGPNMB solution (0.04 µg/g body weight). Levels of key serum markers were measured **(A)** TNF-α, **(B)** SAP, **(C)** NGAL. Each datapoint represents mean ± SD, n = 4 mice per group. **(D)** H&E images of kidney and liver sections harvested from C57Bl/6 mice at week-1, and week-12 post injection.

We expanded the long-term safety assessment of key markers for inflammatory, and tissue function up to day-84 post-treatment. The markers include (a) TNF-a [68], (b) neutrophil gelatinase-associated lipocalin-2 (NGAL), which is produced by the kidneys and a high level will connote possible kidney injury [70, 80] and (c) serum amyloid protein (SAP), which is significantly increased in serum during inflammation [81]. All the markers showed comparable levels (p > 0.05) in the GPNMB-therapeutic treatment groups *versus* saline for days 0, 7 and 84 post-injections (Fig. 6A-C). The trend indicates that there was no long-term damage to the liver and kidneys as well as no induction of inflammatory markers in GPNMB-therapeutic treatment groups. The histological evaluation of tissue sections showed absence of infiltrates or cellular debris (Fig. 6D). Overall, data sets suggest that at a therapeutic effective range of GPNMB therapeutics, there was no induction of inflammation or major organ damage.

### Osteoblast Differentiation and In-vivo Bone Regenerative Assessment

**Fig. 7:**
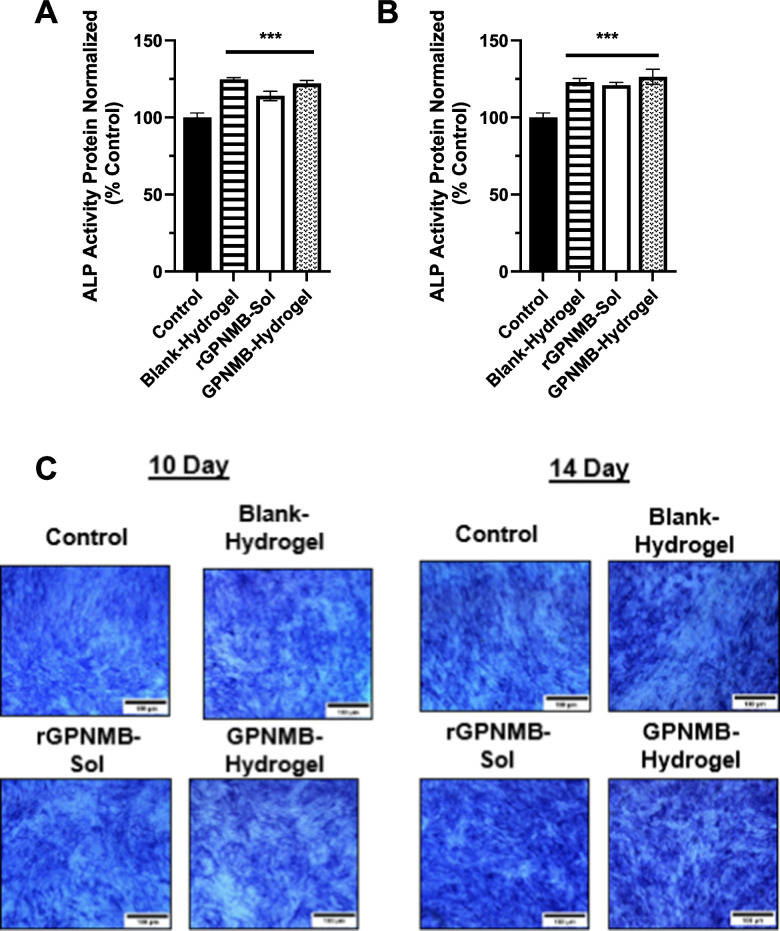
Biofunctional Assessment of GPNMB-hydrogel with Osteoblast Differentiation. Extent of osteoblast (MC3T3-E1) cell differentiation as measured by alkaline phosphatase (ALP) activity on **(A)** day-10 and **(B)** day-14 with **(C)** representative images of ALP staining. Cells received osteogenic media alone (control), osteogenic media in combination with treatments such as blank PLA-b-PEG-b-PLA hydrogels, GPNMB-hydrogels or rGPNMB alone. Each datapoint (mean ± SD, n = 4) is expressed as a percentage ALP activity obtained from cells that received osteogenic media alone (control). ***p < 0.0001 *versus* control cells.

To evaluate the biofunctionality of rGPNMB released from our hydrogel formulations we conducted MC3T3 cell differentiation studies. Studies have shown the effect of rGPNMB protein in enhancing differentiation and subsequent mineral deposition of pre-osteoblast cells, as well as the downstream signaling implications [24–28]. Using control cells, which receive only differentiation media without treatment as a reference, our results demonstrated the ability of GPNMB-hydrogel to support OB differentiation as measured by the level of ALP activity (Fig. 7A-C). It is noteworthy that the blank hydrogel with the osteogenic differentiation media showed potential of facilitating osteoblast differentiation (Fig. 7A-C). Additional studies will be needed to investigate the possible beneficial effects of the PLA-b-PEG-b-PLA hydrogel platform to the osteoblast differentiation. This is important because earlier studies demonstrated that PEG/PLA-based biomaterials when applied in bone regenerative application could promote differentiation of mesenchymal stem cells into osteoblasts [82, 83]. The overall trend from osteoblast differentiation studies demonstrated the biofunctionality of GPNMB protein loaded onto the hydrogel (Fig. 7A-C).

**Fig. 8:**
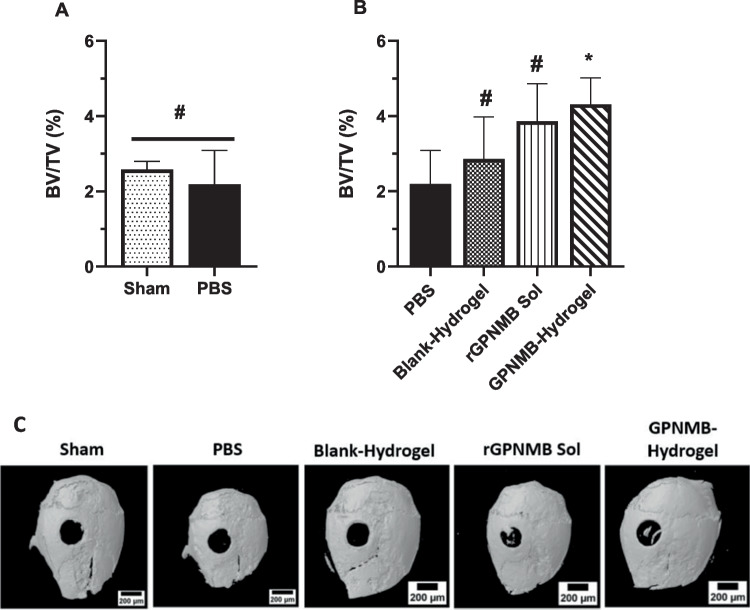
Bone regeneration assessment of GPNMB hydrogel in murine calvarial defect model. **(A-B)** Analysis of new bone formation within defect sites created on the calvaria of mice (C57BL/6) at 5-weeks post-surgery according to different treatment groups Sham, PBS, blank hydrogel, rGPNMB solution and GPNMB hydrogel. Bone formation within the defect is presented as bone volume to tissue ratio (BV/TV%) parameter. **(C)** Representative 3D reconstructed images of the micro-CT analysis of harvested calvaria from sham, blank hydrogel, rGPNMB solution and GPNMB- hydrogel. Each datapoint represents mean ± SD, n = 3–4 mice per group. #p > 0.05, *p < 0.05 *versus* the PBS group.

Critical-sized calvaria defect models are frequently used to assess the efficacy of bone regenerative treatments over time [27, 84]. We encountered a few challenges with the calvaria defect studies pertaining to the very small injection volume of treatment to cover the defect site. To prevent possible run-off of treatments after application, we layered the defect site with absorbable collagen (AC) sponge prior to introducing the treatments- PBS, blank hydrogel and GPNMB-hydrogel (Fig. 8). In the setting, we are expecting the sham group (defect with no treatment and no AC) and PBS (defect with AC) will serve as effective controls (Fig. 8A). The extent of bone regeneration into the defect site was quantified using BV/TV (%) and it shows the values for Sham and PBS groups were comparable (p > 0.05; Fig. 8A) suggesting that the effects of PBS addition to collagen sponge did not play a major role in bone regeneration. For most of the mice/groups, we observed a slight increase in BV/TV (%) within the defect for rGPNMB solution and GPNMB hydrogel, maintained at rGPNMB dose of 0.06 µg/g BW (Fig. 8B). Compared to PBS group, the average value BV/TV (%) increased by 1.8 times and 2 times for rGPNMB solution and GPNMB hydrogel, respectively (Fig. 8B). Representative 3D reconstructed calvarial images for different treatment groups indicate the potential of GPNMB- therapeutics to promote bone growth into the defect area (Fig. 8C). It is important to note that we applied a single dose of rGPNMB of 0.06 µg/g BW for both the rGPNMB solution and GPNMB hydrogel treatment groups. We are of the opinion that additional studies will be needed to overcome the small volume restrictions of the calvaria defects so as to maximize the effective dose that will be applied in the *in-vivo* bone regenerative effects. It may also be beneficial to vary the end point of terminating the experiment post-surgery while assessing *in-vivo* efficacy by varying doses of rGPNMB protein. We could consider alternative models of bone defects to assess the *in-vivo* bone regenerative potential of GPNMB hydrogels.

## Conclusion

The premise for this work is that the clinical relevance of osteogenic therapeutics will benefit greatly from suitable delivery systems. We successfully developed GPNMB hydrogels using a PLA-b-PEG-b-PLA triblock copolymer platform and evaluated its efficacy and safety profiles. We demonstrated the thermoresponsive and viscoelastic features of GPNMB-hydrogel were evaluated using approaches such as (i) dynamic light scattering, (ii) loss and storage moduli, (iii) gelation temperature and time, and (iv) rheological strain and frequency sweep tests. The release of GPNMB protein from hydrogel suggested a diffusion-controlled kinetic which correlated with results from hydrogel degradation studies. We observed excellent *in-vivo* safety profiles based on serum levels of markers of tissue function and inflammation. Additional data suggested there was no ectopic bone formation due to administration of varying doses of GPNMB protein. Overall results from efficacy and safety studies suggested that the PLA-b-PEG-b-PLA thermoresponsive platform could be suitable for GPNMB protein delivery with the possibility of injecting the GPNMB-therapeutics into the defect while relying on gelation at physiological temperature to improve localization at fusion sites and prevent off-target distribution. We consider that *in-vivo* efficacy studies in a fracture model in osteoporotic animal models could highlight unique features of GPNMB-hydrogel pertaining to thermoresponsiveness and injectability.

## Supplementary Information

Below is the link to the electronic supplementary material.

Supplementary file1 (DOCX 1639 KB)

## Data Availability

The data sets generated during and/or analyzed during the current study are available from the corresponding author on reasonable request.
