# Supplementary material for: Fabrication and Evaluation of Thermoresponsive GPNMB-Hydrogels as an Innovative Osteogenic Therapeutic Strategy
Source: Pharm Res. 2025 Nov 18;42(12):2309–27. doi: 10.1007/s11095-025-03978-1 (PMC12754804; doi:10.1007/s11095-025-03978-1)
Supplement: Supplementary file 1 — Supplementary file1 (DOCX 1639 KB) [file 11095_2025_3978_MOESM1_ESM.docx]

**Supplementary Information**

Manuscript Submitted to*:* ***Pharmaceutical Research***

**Fabrication and Evaluation of Thermoresponsive GPNMB-Hydrogels as an Innovative Osteogenic Therapeutic Strategy.**

Tori Czech^1^, Evin Hessel^1^, Jenna Knowles^1^, Kalkedan T. Ameha^1^, Matthew Smith^1,3,4^,

Fayez Safadi^2,3,4^ and Moses O. Oyewumi^1,3^*

^1^Department of Pharmaceutical Sciences, College of Pharmacy, Northeast Ohio Medical University, Rootstown, OH, USA.

^2^Department of Biomedical Sciences, College of Medicine, Northeast Ohio Medical University, Rootstown, OH, USA

^3^UH-NEOMED Faculty Scholar, Northeast Ohio Medical University, Rootstown,

OH, USA

^4^Rebecca D. Considine Research Institute, Akron Children’s Hospital, Akron, OH, USA

*Correspondence:

Moses Oyewumi, B. Pharm, Ph.D.

Professor and Chair, Department of Pharmaceutical Sciences

2025 UH-NEOMED Faculty Scholar

College of Pharmacy, Northeast Ohio Medical University

4209 State Route 44, Rootstown, OH 44272, USA

Tel: 1-330-325-6669; Email: [moyewumi@neomed.edu](mailto:moyewumi@neomed.edu)

**
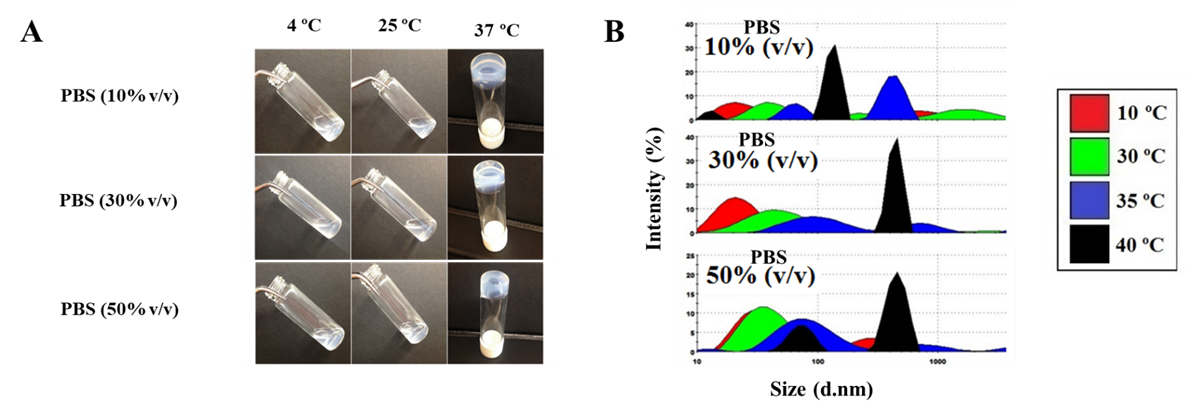
**

**Supplemental Figure S1: Loading Volume Capacity Challenge Using Thermoresponsive Characterizations of the Copolymeric Hydrogel.** **(A)** Photographs of the thermogelling behavior (phase transition observations) of the 20% (w/v) PLA-b-PEG-b-PLA (1500-1500-1500 Da) copolymeric hydrogel with varying volumes of a PBS (10-50% v/v) loaded to observe effect of volume on phase transition at 4, 25, and 37 ºC. We observed phase transition from sol phase at 4 ºC and 25 ºC that transitioned to gel phase at 37 ºC. However, at 30% v/v loading, the structural integrity is affected. **(B)** Representative laser light scattering effects of temperature changes on particle size distribution of 20% w/v copolymeric hydrogels loaded with varying volumes of PBS (10-50% v/v) at varying temperatures.

**
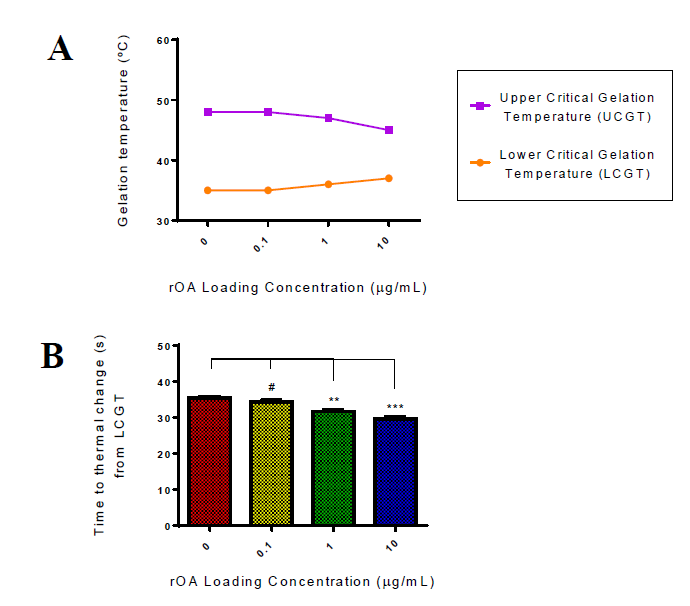
**

**Supplemental Figure S2: Lower Critical Gelation Temperature (LCGT) Determination of GPNMB-Hydrogel (loaded with rGPNMB; rOA) (A)** LCGT and UCGT of 20% w/v PLA-b-PEG-b-PLA (1500-1500-1500 Da) copolymeric hydrogel with variable rOA loading (0, 0.1, 1, 10 μg/mL). **(B)** Time to thermal change for corrected LCGT of the hydrogel at varying rOA(GPNMB protein) loading doses (0, 0.1, 1, and 10 μg/mL), showing 35.3, 34.3, 31.6, and 29.6 s, respectively.

**
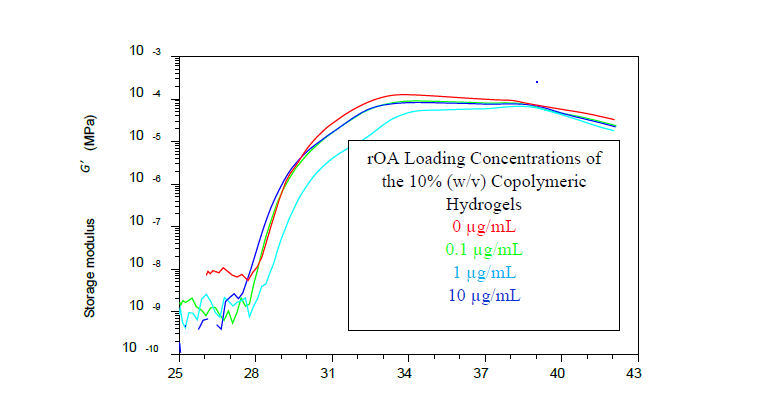
**

**Supplemental Figure S3: Rheological Profile for GPNMB-Hydrogels at various rGPNMB (rOA) loading concentrations.** Representative rheological graphs using a 10% strain rate of a temperature sweep showing the overlaid storage moduli (G’) of the 10% (w/v) PLA-b-PEG-b-PLA (1500-1500-1500 Da) copolymeric hydrogels with varying rOA loading concentrations (0, 0.1, 1, and 10 μg/mL). Storage moduli are virtually superimposable, suggesting protein loading does not negatively affect thermoresponsive behavior of the 10% (w/v) copolymer.


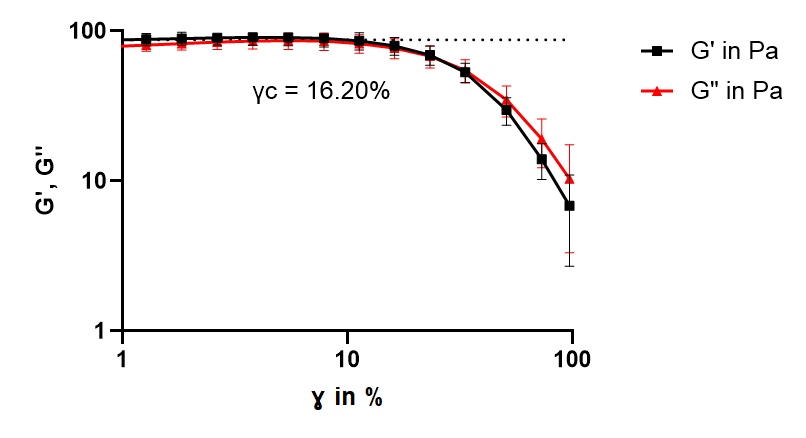


**A**


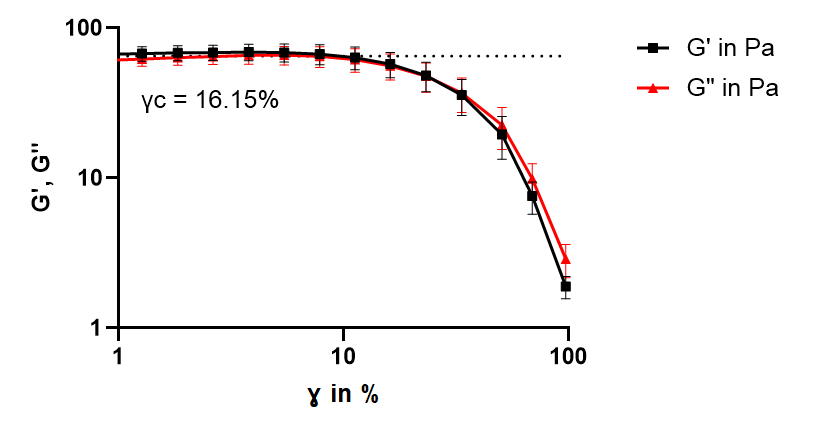


**Supplemental Figure S4: Stain Sweep Tests for (A) blank hydrogel and (B) GPMB-Hydrogel (10 µg/mL).** The critical strain values for blank hydrogel (PLA-b-PEG-b-PLA; 10% w/v) and GPNMB-hydrogel are 16.20% and 16.15% respectively. The critical strain value was derived by finding the first point that deviated beyond 5% from the plateau line of the G’ plot. To do this firstly a plateau line was constructed by using the runs tests to identify the portion of the G’ plot that had a non-significant deviation from linearity, that range was then averaged to find the plateau value. This value is plotted as a dotted line on all strain sweep graphs. The 5% deviation from the plateau line was calculated, and the critical strain of each plot is recorded as the strain value where G’ drops below this 5% value. We conducted the tests using Haake Mars Rheometer (Thermo Fisher Scientific). Other parameters: Loaded Temperature = 20^o^C, Equilibration Temperature 34^o^C, Equilibration time = 50 seconds, Measurement Geometry = P20, Gap = 0.2 mm, Frequency = 0.1Hz and strain oscillation = 0.1-100%. The tests were conducted for three independent blank and GPNMB hydrogel formulations.

**B**

**A**

**
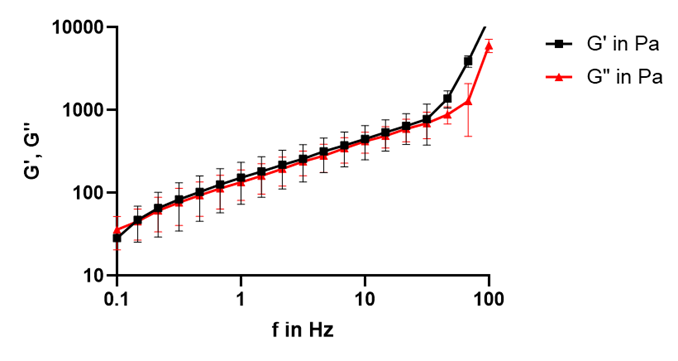
**

**
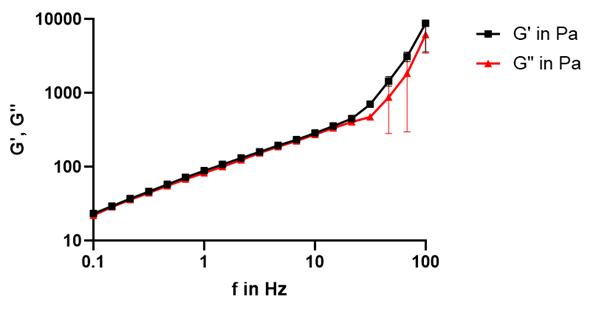
**

**Supplemental Figure S5: Frequency Sweep Tests for (A) blank hydrogel (PLA-b-PEG-b-PLA; 10% w/v) and (B) GPMB-Hydrogel (10 µg/mL).** We conducted the tests using Haake Mars Rheometer (Thermo Fisher Scientific). Other parameters: Loaded Temperature = 20^o^C, Equilibration Temperature 34^o^C, Equilibration time = 50 seconds, Measurement Geometry = P20, Gap = 0.2 mm, Frequency = 0.1-100 Hz and strain oscillation = 0.1%. The tests were conducted for three independent blank and GPNMB hydrogel formulations. The data demonstrated the viscoelastic behavior of the hydrogel with increase in G’ and G’’ across the increase frequency. There was a slight increase in G’ over G” across the frequency sweep test indicating a slight reflection of more of elastic behavior than the viscous behavior across the frequency increase.

**B**


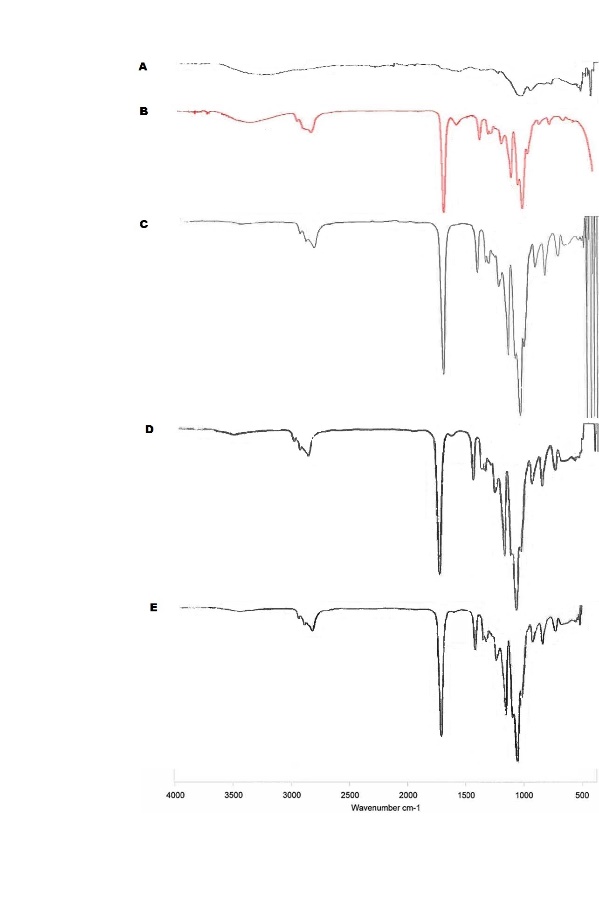


**Supplemental Figure S6:** Representative FTIR spectral analysis of **(A)** rGPNMB protein alone, **(B)** PLA-b-PEG-b-PLA copolymer alone, **(C)** physical mixture of rGPNMB and PLA-b-PEG-b-PLA copolymer, **(D)** Blank PLA-b-PEG-b-PLA hydrogel, and **(E)** GPNMB-Hydrogel (10 µg/mL rGPNMB).


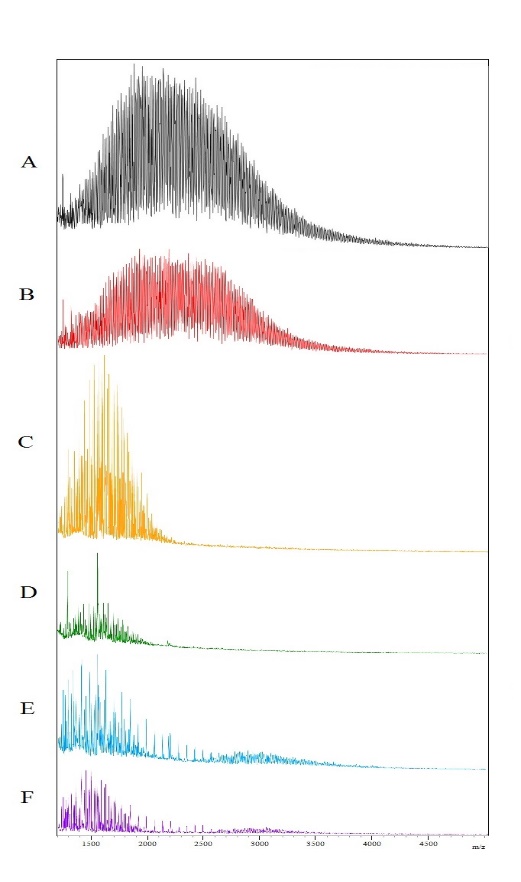


**Supplemental Figure S7: Polymer Degradation of the PLA-PEG-PLA (10% w/v) Hydrogel**. Representative MALDI data from **(A)** PLA-b-PEG-b-PLA co-polymer alone, PLA-b-PEG-b-PLA (10% w/v) Hydrogel on **(B)** the day of preparation and **(C-F)** subjected to degradation for **(C)** 2 weeks after preparation **(D)** 4 weeks after preparation, (E) 6 weeks after preparation and **(F)** 8 weeks after preparation.

**
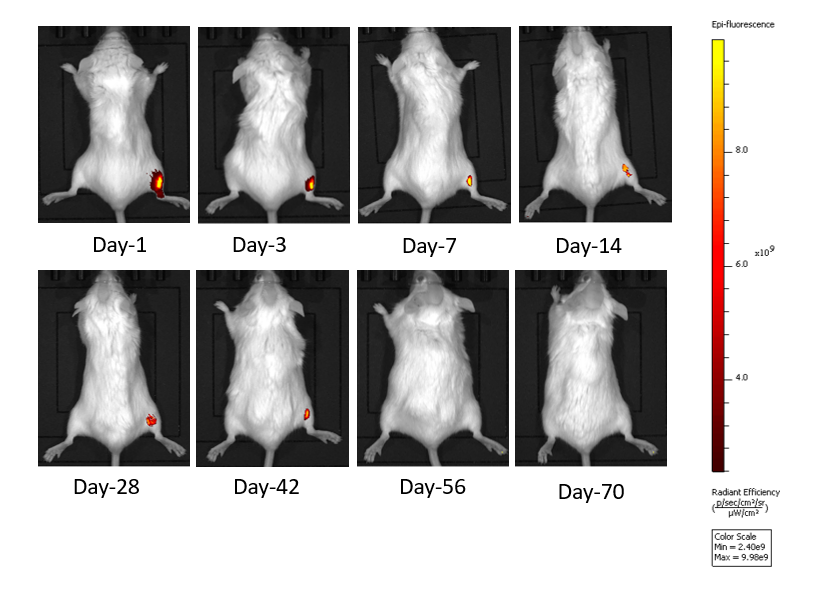
Supplemental Figure S8: Representative Images from Bioretention of Thermoresponsive Hydrogel Prepared with PLA-b-PEG-b-PLA tagged with FKR648 end cap.** Representative images of the total radiant efficiencies after intramuscular injection of the hydrogel over a 10-week period of observation. The results show that the signal was detectable at the earliest time points and decreased gradually over 6 weeks. The signal was lost after 6 weeks.


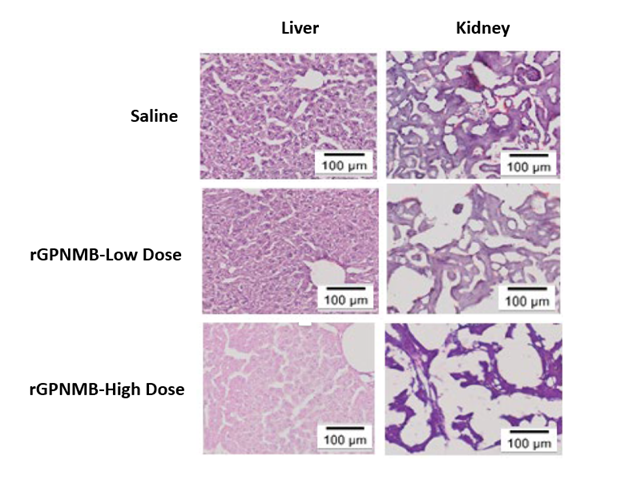


**Supplemental Figure S9: A short-term safety assessment of rGPNMB solution with dose variation showed no detectable signs of tissue damage.** Mice (C57BL/6) were monitored until day 7 post injection after receiving periosseous injections of the following treatments- saline, rGPNMB low dose (0.04 µg/g) and rGPNMB high dose (0.2 µg/g). Representative liver and kidney sections obtained on day-7 post-injection were stained with H &E.

**
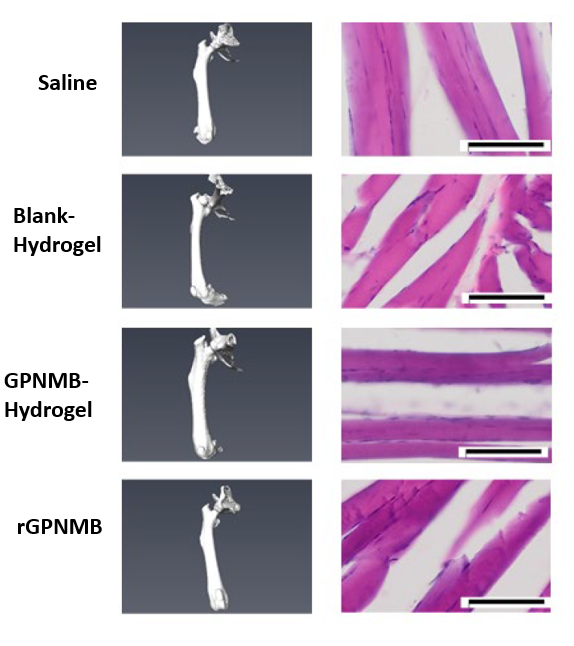
Supplemental Figure S10: GPNMB treatments did not cause detectable ectopic bone formation.** Mice (C57BL/6) were monitored until day 84 post injection after receiving periosseous injections of the following treatments: saline, blank-hydrogel, GPNMB-hydrogel (0.04 µg/g body weight) and rGPNMB solution (0.04 µg/g body weight) 3D-µCT images of whole hindlimbs close to the injection site obtained at day 84 post injection as well as representative H&E images of local injection tissue. The scale bar is 100 µm and representative images show that there is no observable ectopic bone formation present in the tissues for any group.
